# A Target Model Construction Algorithm for Robust Real-Time Mean-Shift Tracking

**DOI:** 10.3390/s141120736

**Published:** 2014-11-03

**Authors:** Yoo-Joo Choi, Yong-Goo Kim

**Affiliations:** Department of Newmedia, Korean German Institute of Technology, 99, Hwagok-ro 61-gil, Gangseo-gu, Seoul 157-930, Korea; E-Mail: yjchoi@kgit.ac.kr

**Keywords:** mean-shift, object tracking, background clutter, asymmetric kernel

## Abstract

Mean-shift tracking has gained more interests, nowadays, aided by its feasibility of real-time and reliable tracker implementation. In order to reduce background clutter interference to mean-shift object tracking, this paper proposes a novel indicator function generation method. The proposed method takes advantage of two ‘*a priori*’ knowledge elements, which are inherent to a kernel support for initializing a target model. Based on the assured background labels, a gradient-based label propagation is performed, resulting in a number of objects differentiated from the background. Then the proposed region growing scheme picks up one largest target object near the center of the kernel support. The grown object region constitutes the proposed indicator function and this allows an exact target model construction for robust mean-shift tracking. Simulation results demonstrate the proposed exact target model could significantly enhance the robustness as well as the accuracy of mean-shift object tracking.

## 1. Introduction

Object tracking, as one of the most fundamental tasks in computer vision, has many applications such as video indexing, automated surveillance, and human computer interaction, *etc*. For this important task, there has been a tremendous amount of research, which is generally categorized into three methodologies, *i.e.*, point-, kernel-, and silhouette-based tracking [1]. Among these approaches, kernel-based tracking has attracted more interest nowadays, thanks to its feasibility of reliable real-time tracker implementation, and this feasibility is known to mainly come from the incorporated mean-shift procedure, helping trackers quickly locate their tracking position to a nearby optimal point.

As an efficient tool for finding the nearest dominant mode of feature space, the mean-shift procedure has been successfully applied to many object tracking algorithms. In [2], Comaniciu *et al.* proposed a color-histogram-based object tracker using the Bhattacharyya similarity measure and Epanechnikov ellipsoidal kernel. They developed a mean-shift target localization scheme, which quickly and reliably optimizes the similarity between the kernel-weighted histogram of a target and that of the candidate region. After this pioneering work, numerous improvements have been reported in the last decade. They are largely schemes using new object representation models [3–5], trackers simultaneously estimating object location as well as kernel scale and orientation [6–8], algorithms based on new similarity measures other than the Bhattacharyya coefficient [9–11], and methods incorporating histogram bin weights for reducing the effect of background clutter [12–14]. Among these, we focus on the problem of background clutter in this paper.

The background clutter problem in kernel-based tracking states that the background colors of a target object inevitably constitute a target representation model. This happens because the kernel shape, which is usually a simple rectangle or an ellipsoid, does not perfectly match the shape of the target object. In order to reduce the effect of such background clutter problems, Commaniciu *et al.* proposed a background weighted histogram method [2], where the target model is modified such that the bins of background colors contribute less to mean-shift optimization. Unfortunately, such an effort to eliminate the dominant background features from the target model is proven to not affect the target localization process, because the same background weights were also incorporated into the modification of the candidate histogram. Ning *et al.* pointed out this problem in [12] and proposed the use of the background weights only for target model construction, which they called a corrected background-weighted histogram method. Moreover, in [13], Li and Feng proposed an adaptive kernel method, where the background weights developed in [2] were also used to further adapt the kernel. In effect, this adaptive kernel method is supposed to modify only the target model, as was the case of [12], but using different bin weights (*i.e.*, squares of the weights used in [12]). Instead of using such background color probability, Jeyakar *et al.* employed a likelihood of object color to modify the target representation model [14]. By defining the likelihood as the log of the ratio between the color distributions of inside and outside the kernel region, they designed two separate weights for modifying the target and candidate models, respectively.

All these schemes demonstrated more reliable tracking results by appropriately modifying the target histogram, but such a histogram weighting method itself seems to be an intuitive and indirect method in the sense of background elimination from a target model. For more direct and strict background elimination, an indicator function in an asymmetric kernel could be exploited [7,15]. Since the indicator function directly specifies whether a pixel belongs to the target object or not, background pixels within a kernel can be strictly excluded from the target model. However, to the best knowledge of the authors, it has not been detailed so far how such an indicator function could be created and what amount of gains could be achieved by incorporating the function. Usually the indicator function has been assumed given perfectly from another stage of application, but this assumption is generally far from reality and such object detection is just another challenging problem.

In order to reduce the interference of background clutter, this paper proposes a novel indicator function generation method and demonstrates an exact target model construction that could significantly enhance the robustness as well as the accuracy of kernel-based tracking results. The proposed method takes advantage of two ‘*a priori*’ knowledge elements, which are inherent to a kernel support for initializing a target model. More specifically, based on the ‘*a priori*’ knowledge that the outside of an initial kernel is background, background labels are propagated to inside the kernel, and then by considering the ‘*a priori*’ knowledge that there is only one target object near the center of given kernel, the proposed algorithm grows a region using an object (foreground) seed near the kernel center. The grown region constitutes the proposed indicator function and this allows an exact target model construction for robust mean-shift object tracking.

The rest of this paper is organized as follows: Section 2 explains the conventional method of mean-shift object tracking with various previous background weighting algorithms to explain how an indicator function is used for constructing the target model of the proposed tracking scheme. Then, in Section 3, we describe the proposed method for generating the indicator function, where the proposed gradient-based label propagation and foreground region merging methods are detailed. Simulations for an extensive comparison study and their results are presented in Section 4. Finally, we conclude this paper in Section 5.

## 2. Mean-Shift Object Tracking

In the conventional mean-shift object tracking, the tracking position *x* is updated recursively to the next position *x′* by:

(1) $$x^{'}=x+\Delta x$$

where:

(2) $$\Delta x=\frac{\sum_{i}g\left(x_{i}-x\right)w(x_{i})(x_{i}-x)}{\sum_{i}g\left(x_{i}-x\right)w(x_{i})}$$

In Equation (2)*g* (·) is the shadow of the kernel profile *k*(*x*), (*i.e.*, g(x) = −*k*′(*x*) ) and *w*(*x_i_*) is the back-projection weight, given as:

(3) $$w\left(x_{i}\right)=\sum_{u=1}^{m}\sqrt{\frac{q_{u}}{p_{u}}}\delta \left[b\left(x_{i}\right)-u\right]$$

where δ[·] is the Kronecker delta function, *b*(*x_i_*) is the bin number associated with the pixel location *x_i_*, and *m* is the number of bins of target (candidate) histogram. *q_u_* and *p_u_* denote the *u*-th bins of target and that of candidate histogram, respectively, which are defined by:

(4) $$q_{u}=\sum_{i=1}^{n_{t}}d_{t}\left(u,x_{i}\right)k(x_{i}-x_{t})\delta (b\left(x_{i}\right)-u]$$

(5) $$p_{u}=\sum_{i=1}^{n_{c}}d_{c}\left(u,x_{i}\right)k(x_{i}-x_{c})\delta (b\left(x_{i}\right)-u]$$

where *n_t_* and *n_c_* denote the numbers of pixels in target and candidate regions. x*_t_* and *x_c_* are the centers of the target and candidate regions. *d_t_*(*u*,*x_i_*) and *d_c_*(*u*,*x_i_*) are the weights for the *u*th bin and the pixel location *x_i_* of the target and the candidate histogram, respectively.

**Figure 1.**
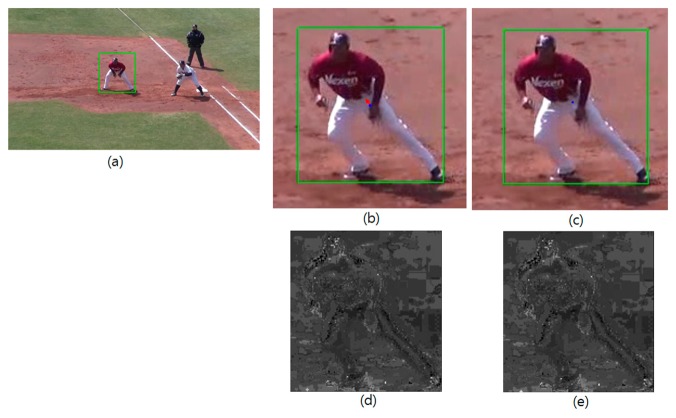
An example of mean-shift tracking refinement: (**a**) video frame at time t with the kernel support for target, (**b**) a zoomed part of video frame at time t + 1 with initial candidate kernel support and the first refinement result, (**c**) a zoomed part at time t + 1 with shifted candidate kernel support after the first refinement and the second refinement result, (**d**) the back-projection weights for the first refinement, and (**e**) the back-projection weights for the second refinement, respectively.

Figure 1 shows an example of the above iterative tracking result with the weights of *d_t_*(*u*,*x_i_*) = *d_c_*(*u*,*x_i_*) = 1 for all *u* and *x_i_*. Figure 1a,b show the video frames at time *t* and *t* + 1 with green boxes indicating the regions of support for the kernel k(·) in Equations (4) and (5). The blue dot in the green box of Figure 1b is the starting tracking position *x* in Equation (1), and the red arrow shows Δ*x*. From Equation (2), this refinement is supposed to be done by finding the gravity center of *w*(*x_i_*) and Figure 1d,e show the images of this back-projection weight (see Equation (3)) for the first two iterations. In these images, brighter pixels represent the higher values of *w*(*x_i_*), and thus the mean-shift refinement of Equation (1) pushes the tracking position *x* to this brighter part of the image *via* Equation (2). As can be seen from the figures, for reliable mean-shift tracking, a large portion of the brighter pixels should belong to the baseball player, which is the target object to be tracked. This proposition can be easily understood by taking the simple example of a synthesized video having a red ball moving against blue background. Let us assume that we track the red ball and a tight kernel window including the red ball is given. In the following frame, the tracking procedure starts at the position of the kernel window of the previous frame, and thus only a part of the moving red ball is included in the kernel window. This situation makes the target and the candidate histogram models such that *q_u_* > *p_u_* when *u* denotes red color and *vice versa* for blue color. This situation results in high back-projection weight, *w*(*x_i_*), for the red ball and low projection weight for background via Equation (3). This high back-projection weight on the target object pushes the tracking position to the red ball until the ball is fully belonging to the kernel window. However, if we assume a red spot is on the blue background, the refinement of tracking position stops when the number of red pixels in the kernel window reaches to the number of pixels of the red ball area even though the kernel window does not fully include the red ball. This perturbs the accurate tracking and the amount of perturbation depends on the size of red spot on the blue background. According to the same principle, the brighter clutters outside the target object in Figure 1d,e bring about the perturbation of tracking result via the gravity center calculation by Equation (2). In order to reduce this background clutter interference, the background weights of *d_t_*(·,·) and *d_c_*(·,·) have been designed in several ways. After getting the normalized histogram, {*g_u_*}*_u_*_=1,…,_*_m_* of the neighboring pixels of kernel support (*i.e.*, the neighboring outside region of the green box in Figure 1a, Comaniciu *et al.* set up the background weights in [2] such as:

(6) $$d_{t}\left(u,x_{i}\right)=d_{c}\left(u,x_{i}\right)=\min\left(g_{s}/g_{u},1\right)$$

where *g_s_* is the smallest nonzero value of *g_u_*. Note that the background histogram of target region (*i.e.*,{*g_u_*}*_u_*_=1,…,_*_m_*) is also used for setting up *d_c_*(u,*x_i_*) (the background weights for candidate). Although this usage is based on a general assumption that the background histogram is not changed severely within a few video frame interval, the same weights in the numerator and the denominator part of the back-projection weight (see Equation (3)) cancel each other's effects out in the mean-shift procedure. By noting this problem, Ning *et al.* used only *d_t_*(u,*x_i_*) of Equation (6), while leaving *d_c_*(u,*x_i_*) = 1 for all *u* and *x_i_* in [12], and Li and Feng used this background histogram not only for *d_t_*(u,*x_i_*) and *d_c_*(u,*x_i_*), but also for further modifying the back-projection weight such as [13]:

(7) $$w^{'}\left(x_{i}\right)=\sum_{u=1}^{m}\min(g_{s}/g_{u},\;1)\;\times \;\sqrt{\frac{q_{u}}{p_{u}}}\delta [b\left(x_{i}\right)-u]$$

This weighting scheme, however, can be replaced by using the weights of:

(8) $$d_{t}\left(u,x_{i}\right)={\left[\min\left(g_{s}/g_{u},1\right)\right]}^{2}\;\text{and}\;d_{c}\left(u,x_{i}\right)=1$$

with the calculation of back-projection weight by Equation (3), because the same weights, d_t_(·,·) = dc(·,·), for *q_u_* and *p_u_* have no effect on the mean-shift procedure of Equation (2).

Instead of designing the weights *d_t_*(·,·) and *d_c_*(·,·) based only on the background histogram, Jeyakar *et al.* incorporated the foreground histogram also to define such weights in [14]. They defined a likelihood that a particular color belongs to foreground or background such as:

(9) $$L_{t}\left(u\right)=\log\frac{\max(q_{u},\in )}{\max(g_{u},\in )}\;\text{and}\;L_{c}\left(u\right)=\log\frac{\max(p_{u},\in )}{\max\left(h_{u},\in \right)\;}$$

where {*h_u_*}*_u=1,…,m_* is the normalized histogram of the neighboring background of the candidate kernel support (*i.e.*, the neighboring outside region of the green box in Figure 1b,c). Then, by using the likelihood, they proposed the weights:

(10) $$d_{\text{X}}\left(u,x_{i}\right)=\max\left(1-\frac{1}{1+\exp\left\{-\frac{L_{x}\left(u\right)-a}{b}\right\}},\;0.1\;\right)$$

where *X* is one of *t* and *c* for target and candidate models, respectively, and *a* and *b* are the control parameters, of which typical values are all 1.

In contrast to the above conventional background weights, the proposed scheme employs a position dependent weight for target model construction such as:

(11) $$d_{\text{X}}\left(u,x_{i}\right)=d\left(u\right)\times \text{I}\left(x_{i}\right)$$

where *d*(*u*) denotes bin weights, which can be any of the above background weights including the case of always 1, and I(*x_i_*) is the indicator function, specifying whether the pixel location *x_i_* belongs to the target object or not. As for the candidate background weights, we set *d_c_*(*u*,*x_i_* = 1) for all *u* and *x_i_*, under the considerations of object deformation and the uncertainty of the candidate background region.

## 3. Generation of Indicator Function

In many object tracking applications, kernel support for the target model is usually initialized by user interaction or given by an object detector as the form of a simple region. This region can be assumed, without loss of generality, larger than the target object and containing one such target object near its center. These assumptions translate the problem of indicator function generation into a bi-label segmentation problem given the two ‘*a priori*’ knowledge elements: (1) outside of the region is background for sure; and (2) one target object is located near the center of the region. Hence, we first initialize the indicator function I(*x_i_*) such that pixels in outside of the region are marked as ‘background’:

(12) $$I\left(x_{i}\right)=\{\begin{array}{c} -1\;\text{when}\;x_{i}\in R\\ 0\;\text{otherwise} \end{array}$$

where *R* denotes the region for the target model. Then, this set of initial background labels is propagated into the region *R*, so as to leave possibly one or multiple candidate target objects within the region. After completing this background label propagation, a region growing is performed to find one largest connected region near the center of *R*, resulting in the proposed indicator function.

### 3.1. Gradient-Based Background Label Propagation

Basically, the proposed background label propagation is performed by iteratively investigating the pixels contiguous to the border of the background region. This border is defined by the set *B* of background pixels, which are neighbors of unsettled pixels, such as:

(13) $$B=\left\{x_{i}|I\left(x_{i}\right)=0\;\text{and}\;\sum_{x_{j}\in N_{4}(x_{i})}I\left(x_{j}\right)<0\right\}$$

where *N*_4_(*x_i_*) is the 4 neighbors of *x_i_*. Then, for each unsettled pixel *x_k_*(*i.e.*,*I*(*x_k_*) = −1), which is adjacent to the set *B* (A pixel ***x****_k_* is said to be adjacent to a set *B*, when *B* contains one or more pixels contiguous to ***x****_k_*), we find the most similar neighboring pixel by:

(14) $$x_{j}^{*}=\arg\min_{x_{j}\in N(x_{k})}\left|G(x_{k})-G(x_{j})\right|$$

where *N*(*x_k_*) is the set of the neighboring background pixels of *x_k_*, defined by:

(15) $$\text{N}\left(x_{k}\right)=\left\{x_{j}|x_{j}\in N_{4}\left(x_{k}\right)\;\text{and}\;I\left(x_{j}\right)=0\right\}$$

And G(*x_k_*) in Equation (14) means the magnitude of gradient, defined by:

(16) $$\text{G}\left(x_{k}\right)=\sqrt{{\left(i\left(x_{k}\right)-i(x_{k}+u_{x})\right)}^{2}+{\left(i\left(x_{k}\right)-i(x_{k}+u_{y})\right)}^{2}}$$

where *i*(·) denotes the intensity of a pixel, and *u_x_* and *u_y_* are the unit vectors in the *x* and *y* directions, respectively. Then, the pixel *x_k_* is to be classified as ‘background’ when *G*(*x_k_*) and $\text{G}(x_{j}^{*})$ are close enough with each other, *i.e.*:

(17) $$I\left(x_{k}\right)=\{\begin{array}{c} 0\;\text{when}\;\left|G\left(x_{k}\right)-G(x_{j}^{*})\right|[\tau \\ 1\;\text{otherwise}\; \end{array}$$

where *τ* is the threshold of the proposed label propagation (although the threshold *τ* was selected manually in this paper, this type of gradient threshold has been widely studied in the image segmentation field, and readers may refer to [16] for automatic control of this parameter.).

Once all the unsettled pixels adjacent to the set *B* have been investigated, the set *B* becomes renewed by accommodating only the newly found ‘background’ pixels, and then the label propagation via Equations (14) and (17) is repeated. This propagation and renew process is to be iterated until the set *B* is renewed as an empty set, resulting in a few of unsettled regions encompassed by the pixels having *I*(*x_i_*) = 1.

**Figure 2.**
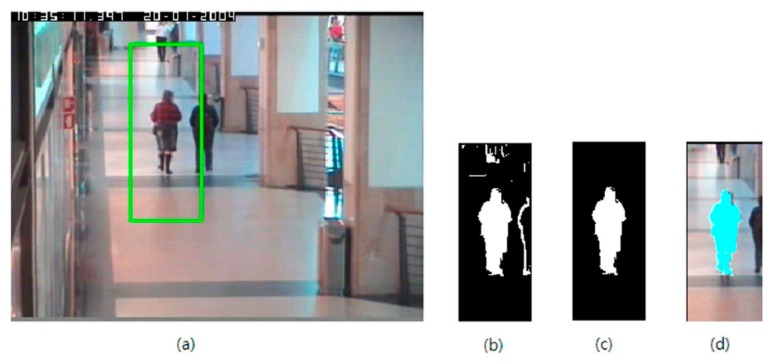
An example of the proposed gradient-based background label propagation and the region growing result: (**a**) a video frame at time *t* with the kernel support for the target, (**b**) gradient-based background label propagation result, (**c**) foreground region growing result, and (**d**) the extracted target object (cyan color) superimposed on the image of kernel support region.

As the result of this gradient-based background propagation, candidate target objects within the region *R* will be represented by the set of pixels having *I*(*x_i_*) = 1 for contour pixels and *I*(*x_i_*) = −1 for inner pixels. Especially, if an object was separated by a given kernel (*i.e.*, the region *R*), the object will be remained as a thin contour line consisting of pixels having *I*(*x_i_*) = 1. Figure 2b shows an example of this background label propagation result. As explained before, target object near the center of *R* is represented as a large connected area (the pixels of *I*(*x_i_*) = 1 or *I*(*x_i_*) = −1 are depicted as white in the figure) and the pedestrian separated by given kernel support (the green box in Figure 2a) is shown as a thin line. The clutter in the off-center area of Figure 2b comes from the high intensity activities of edge pixels, and will be eliminated from the proposed indicator function by the following region growing method.

### 3.2. Region Growing

Region growing is a procedure that assigns pixels to a region based on the predefined criteria of growth. The approach starts basically with a set of ‘*seed*’ pixels, and the set grows by recursively appending similar neighboring pixels. In our method, the set is initialized with a single pixel, which was not background and is near the kernel center. Then the set grows to a large connected foreground region which is a candidate for the target object. After completing the region growing, the proposed scheme investigates pixels in the kernel center area to find any remaining pixels which are not background and not assigned to a foreground region yet. If such pixel was found, region growing is conducted again using the detected seed pixel, and this seed exploration and region growing process is repeated until no more seed pixels can be found. This repeated region growing may produce multiple foreground regions, and thus we choose the largest one as the proposed target object based on the ‘*a priori*’ knowledge that only one target object is located near the center of a given kernel support.

Now, in order to formally describe the proposed region growing algorithm, let us define *C* be the small (in this paper, for simulations, we set the size of *C* become one ninth of the given kernel support area) rectangular region co-centered with the kernel support. After selecting a pixel *x_s_* from *C*, of which label is *I*(*x_s_*) = 1 or *I*(*x_s_*) = −1, two sets *S*^0^ and *O*^0^ are initialized by:

(18) $$S^{0}=\left\{x_{s}\right\}\;\text{and}\;O^{0}=\left\{x_{s}\right\}$$

where *S*^0^ and *O*^0^ are the initial sets for seed and object pixels, respectively. Then, the proposed region growing is performed by appending pixels according to the following criteria:

(19) $$S^{k}={\left\{x_{j}|x_{j}\in N_{8}\left(x_{i}\right),\;\left(I\left(x_{j}\right)=1\;or\;I\left(x_{j}\right)=-1\right),\;\text{and}\;x_{i}\in S^{k-1}\right\}}_{,k=1,2,\ldots }$$

and the set of object pixels is updated by:

(20) $$O^{k}=O^{k-1}\cup S^{k}$$

where *N*_8_(*x_i_*) is the eight neighbors of *x_i_*. Note here that the proposed region growing resorts to an eight neighbors system to merge the parts of an object which are connected by a thin line, while the proposed background label propagation employed a four neighbors system to leave the inner parts of an object intact. As *k* increases, this iterative procedure gathers more object pixels until the set *S^k^* becomes empty. After completion, an identifier is given to the grown object pixels by:

(21) $$I\left(x_{j}\right)=l,\;\text{for}\;x_{j}\in O^{K}$$

where *l* is the initial value for object identifier and the iterative region growing was assumed completed at stage *K*.

Once this identifier allocation has been done, we increase the object identifier *l* by 1, and then check any new seed pixel *x_s_* from *C* again. Note, because of the above identifier allocation, that no more seed pixels can be found when there exists only one object near the center of the given kernel. However, once a new seed pixel was found, the above region growing (*i.e.*, the Equations (18)–(20)) shall be performed again and the increased identifier *l* + 1 will be allocated to the newly grown object pixels. This identifier increment, seed pixel exploration, region growing and identifier allocation will be repeated until no more seed pixels can be found from *C*.

Finally, once all the foreground pixels in *C* were processed, we choose only one target object by:

(22) $$l^{*}=\arg\max_{L\in \{l,l+1,\ldots \}}{\left|I(x_{j})\right|}_{L}$$

where |·|*_L_* is the cardinality of the set of pixels having identifier value of *L*.

Now the proposed indicator function will be generated by:

(23) $$I\left(x_{i}\right)=\{\begin{array}{c} 1\;\text{when}\;I\left(x_{i}\right)=l^{*}\\ 0\;\text{otherwise} \end{array}$$

Figure 2 shows a result of the proposed indicator function generation. Given kernel support by the green box of Figure 2a, the proposed background label propagation gives the result of Figure 2b. After performing the above explained region growing algorithm, only the pedestrian shown near the kernel center remains as in Figure 2c. This target object was superimposed on the image of the kernel support shown as a cyan color area in Figure 2d.

## 4. Simulation Results

In order to show the feasibility of the proposed scheme, we implemented the previous background clutter reduction algorithms which were explained in Section 2. The implemented bin weights will be denoted by A1 (Algorithm 1 of Commaniciu *et al.*'s [2]), A2 (Algorithm 2 of Ning *et al.*'s [12]), A3 (Algorithm 3 of Jeyaka *et al.*'s [14]), and A4 (Algorithm 4 of Li *et al.*'s [13]) method, respectively. More specifically, A1 set up the background weights *d_t_*(*u*,*x_i_*) and *d_c_*(*u*,*x_i_*) using Equation (6), while A2 used the same *d_t_*(*u*,*x_i_*) as that of A1, but set *d_c_*(*u*,*x_i_*) = 1 for all *u* and *x_i_*. A3 and A4 used Equations (10) and (8), respectively, for the computation of such background weights. Then, each of the background weights in the methods A1, A2, A3 and A4 is combined with the proposed indicator function via Equation (11), resulting in the position as well as the histogram bin dependent weights. We denote these combined methods by P1, P2, P3 and P4, (*i.e.*, Px combines the bin weights of Ax with the proposed indicator function, where x = 1,2,3 and 4.) In other words, the proposed methods set the bin weights for target model by multiplying *d_t_*(*u*,*x_i_*) of A1, A2, A3 and A4 by I(*x_i_*) of Equation (23) and set *dc*(*u*,*x_i_*) = 1 for all *u* and *x_i_*. We investigated the tracking errors of each tracker (with and without the proposed indicator function). Here, the tracking error is defined by the distance from the center of the ground truth object to the tracked object (or kernel) center for each video frame.

**Figure 3.**
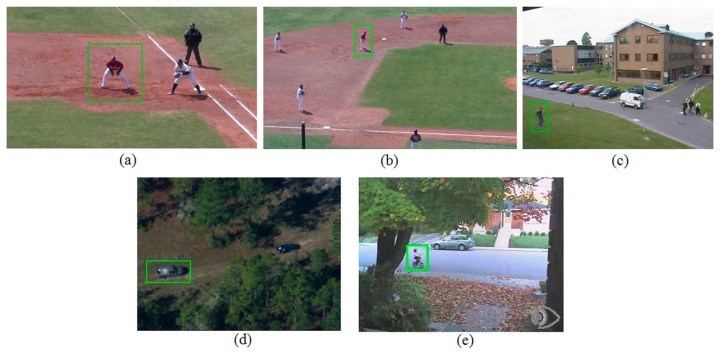
Representative image with target object (green box) for the test sequence of (**a**) Baseball-1, (**b**) Baseball-2, (**c**) Campus, (**d**) EgTest05, and (**e**) Bike.

To conduct such comparative tests, we carefully selected five video sequences, which have distinctive characteristics in the amount of scale change, deformation, and the occlusion of the target object. Three of the selected videos (Campus, Egtest05, and Bike) are publically available and can be obtained from the Performance Evaluation of Tracking System (PETS) [17,18] and the OpenCV Cookbook datasets [19], and the rest of them (Baseball-1 and Baseball-2) were produced by the authors using a Sony CX560 camcorder. The properties of each test video sequence are summarized in Table 1 and each representative image with target object is illustrated in Figure 3. The numbers between the parentheses in the ‘image size’ column of Table 1 represent the numbers of video frames used for this simulation.

**Figure 4.**
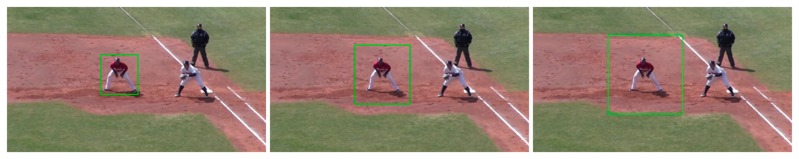
Three different sizes of kernel support for the test sequence Baseball-1.

For all tests, Red, Green and Blue (RGB) color space was employed to create a histogram, which was quantized into 16 × 16 × 16 bins for realtime implementation. The kernel for the target object was set with the Epanechnikov profile [2] as having three different sizes, where the smallest size was the tight fit to the target object, and the other two were set as 1.3 times and 1.7 times as large as the size of the smallest kernel in the width and in the height, respectively. This different size of kernel support is to simulate the imperfect initialization of target specification and to study the effect of background clutter according to this imperfection. Note that, in some applications, the initial kernel is usually given by user interaction via equipment such as mice and tablets, *etc.* Figure 4 shows these three sizes of kernel support for the test video ‘Baseball-1’.

In this figure, we can identify that background colors (outside the kernel support) could be varied significantly by the choice of kernel size, that means the performance of the algorithms A1, A2, A3, and A4 (*i.e.*, the tracking schemes employing background histogram) could be seriously influenced by this kernel size.

Table 2 summarizes the simulation results, where each number represents the tracking error averaged over all the tested frames of each video. Also, in the table, bold numbers indicate the best tracking results for each row (*i.e.*, for a given kernel size and test sequence), (F)s next to the tracking error mean the cases of tracking failure, and the floating point numbers in brackets (*i.e.*, [11.5]) denote the Accuracy Improvement (AI) ratios, which were computed by:

(24) $$AI=\frac{E_{A}-E_{P}}{E_{P}}\times 1$$

where *E_A_* and *E_P_* are the averaged tracking errors before and after applying the proposed indicator function, respectively.

First of all, from the table, we can see that the application of the proposed indicator function always improves the tracking performance except for the two cases of P2 with small kernel size and P4 with normal kernel size for the test sequence Baseball-2. However, the difference of tracking errors is far less than 1 pixel (0.3 for small kernel case and 0.1 for normal kernel case). On the other hand, the benefit of the proposed indicator function is observed to be substantial such that the largest gain reaches up to 56.6% of AI (in the case of P2 with large size kernel for the test sequence Campus) and up to 19.7 pixels (in the case of P1 with large size kernel for the test sequence EgTest05), even without considering tracking failure cases. For the tracking failure cases, the AIs in the table are generally less meaningful because kernel support is usually stuck in some background part where tracking failure happened and thus higher AIs do not always imply better tracking performance (nevertheless, positive AIs of these failure cases usually imply that the proposed method is able to track the given target object for more video frames). Instead, to show the robustness improvement by the proposed scheme, we counted the number of cases where tracking failure of the previous algorithms has been overcome by the proposed indicator function. More specifically, we define the Robustness Improvement (RI) ratio as:

(25) $$RI=\frac{F_{A}-F_{P}}{T}\times 100$$

where *F_A_* and *F_P_* are the numbers of tracking failure cases of the previous and the corresponding proposed trackers, respectively, and *T* is the number of total trials (*i.e.*, 15 in our case for each tracker). Table 3 summarizes these RIs and the averaged AIs for each proposed scheme. Here, the average has been computed for all 15 cases, but without the tracking failure cases (*i.e.*, the AIs with (F) in the columns of P1 and P3 of Table 2). Hence the average *AI* represents the gain when the previous and the proposed methods successfully track the given target object, while RI shows the gain when the previous method fails to (but the proposed scheme successfully) track(s) the object.

**Figure 5.**
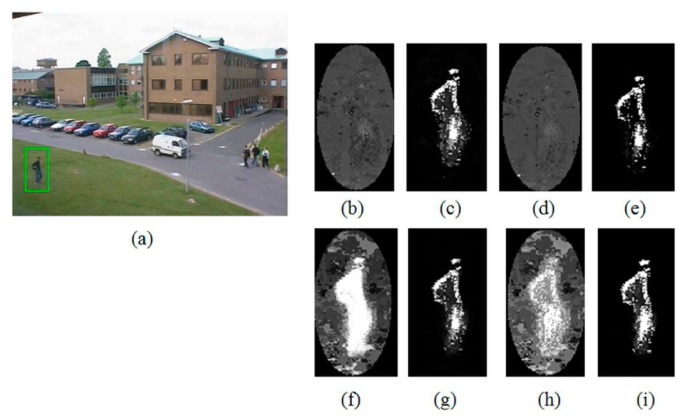
Back-projection weight images for (**a**) the first frame of Campus test sequence in the algorithms of (**b**) A1, (**c**) A2, (**d**) A3, (**e**) A4, (**f**) P1, (**g**) P2, (**h**) P3, and (**i**) P4.

As can be seen from the table, the proposed indicator function significantly improves the tracking robustness as well as accuracy. Especially, in the cases of P1 and P3, the ratios of fixed tracking failure reached 40% of the total trial, and in the case of P1 tracking accuracy has been improved by about 30% on average. Here, the performance gain of the proposed indicator function is observed to be relatively higher in P1 and P3. As can be seen from the Table 2, this is because A2 and A4 are relatively more accurate and robust than the methods A1 and A3. To be more specific about the rationale of this different performance gain, we analyzed the changes of back-projection weight (*i.e.*, *w*(*x_i_*) in Equation (3)) before and after applying the proposed indicator function. This is important because, as can be seen from Equation (2), the amount of position update for tracking eventually corresponds to the weighted average of pixel coordinates based on this weight. Figure 5 shows the back-projection weight of each algorithm for the first frame of the Campus sequence, where brighter pixels mean higher back-projection weight values. As can be seen from the figure, the back-projection weights of A2 and A4 have more bright pixels on the target object, and this explains the more accurate and robust tracking performance of A2 and A4 compared with that of A1 and A3. Similarly, the changes of back-projection weight from Figure 5b,d to Figure 5f,h explain the higher gains of P1 and P3 by applying the proposed indicator function. On the other hand, in the case of Figure 5g,i, only slight changes from Figure 5c,e can be observed, and this accounts for the relatively small gains of 4.1 pixels and 1.1 pixels in accuracy improvement for P2 and P4, respectively. These only slight changes of back-projection weight are due to the fact that the colors of the background area (*i.e.*, the outside region of the kernel support) are very similar to those inside the given kernel, and thus the background clutter reduction methods of A2 and A4 were able to successfully suppress the effect of such background colors. However, this situation is not always guaranteed, and one serious exception case can be found in the test sequence Bike.

**Figure 6.**
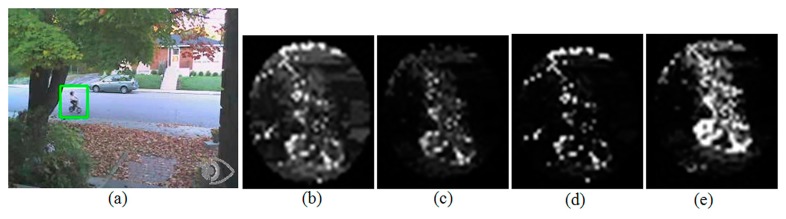
Back-projection weight images for (**a**) the first frame of Bike test sequence in the algorithms of (**b**) A2, (**c**) P2, (**d**) A4, and (**e**) P4.

**Figure 7.**
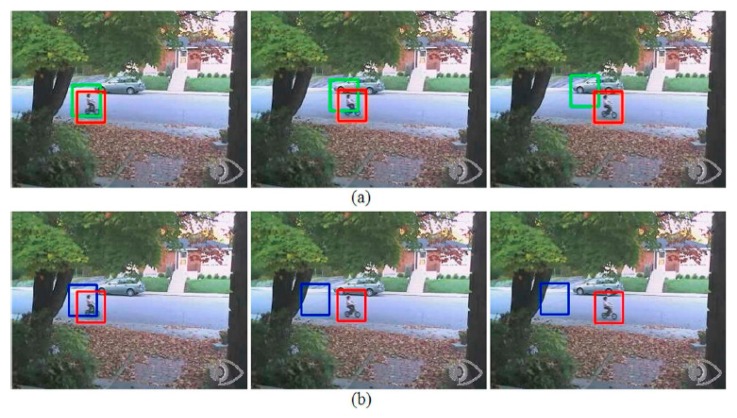
Tracking results of the test sequence Bike by the algorithms (**a**) A2 (green box) and P2 (red box), and (**b**) A4 (blue box) and P4 (red box). Each three figures are the 55th, the 62nd, and the 69th frames from left to right.

Figure 6 shows the changes of back-projection weight from A2 to P2 and from A4 to P4. In this case, the bright white pixels at the upper part of the given kernel are hardly eliminated from the back-projection weight image because the color is very similar to the upper part of the given target object but there are no such color outside the given kernel. Thus the bright pixels at the upper part of Figures 6b,d push the tracked position of A2 and A4 upward, resulting in tracking failure for all kernel sizes. However, in the case of Figure 6c, we can identify these bright pixels at the upper part are successfully removed, and in the case of Figure 6e, many more white pixels are observed on the target object. These changes help the schemes P2 and P4 successfully track the given target object until the end of the test sequence Bike. Figure 7 shows the tracking results of the test sequence Bike when the algorithms A2 and A4 start to fail their tracking.

Finally, in order to identify the computational complexity of the proposed indicator function, we measured the execution time of each algorithm. For more specific analysis of the complexity, each tracking algorithm is regarded as being comprised of three stages, (*i.e.*, target model construction, target candidate model construction and new centroid computation stages). Then we measured the execution time of each stage for each algorithm with the iteration number of centroid computation. In order to eliminate the influence of operating system, all the five test sequences with three different sizes of kernel supports (*i.e.*, 15 cases in total) were tested for the initial 40 frames of each test sequence, and we averaged all the measured times for each processing stage. All the measurement were done on the same system using the Windows 7 operating system with an Intel Core i7 2.67GHz CPU and 4 GB RAM.

Table 4 summarizes the experimental results. As can be seen from the table, the average time for target model construction was increased by about 65.1%, but since the execution time for tracking is longer than the target model construction, this complexity increase corresponds to only about 19% of average total execution time per frame. Moreover, if we consider the target model construction is done for once at the initialization step, the added computation to track the whole 40 frames becomes only about 0.47%. With the average execution times in Table 4, even though the target model construction should be done for every video frame, the trackers using the proposed indicator function (*i.e.*, P1, P2, P3 and P4) are able to track 304.3 frames per second on average.

## 5. Conclusions

Mean-shift tracking is a well-known practical method to reliably track an irregular object in real-time. For more accurate and robust performance of such mean-shift tracking, this paper introduced a target model construction based on an indicator function and provided an algorithm to create the indicator function. After formulating the generation of the indicator function as a two-label segmentation problem, we developed a method of gradient-based background-label propagation and region growing. Using two ‘*a priori*’ knowledge elements inherent to an initial kernel, the developed method extracts an object segment from the given kernel and this segmented region constitutes the proposed indicator function. This proposed method to generate the indicator function is performed once in the whole tracking procedure, its required computations do not affect the real-time implementation of the mean-shift tracker once the tracker without indicator function is fast enough in itself.

The proposed indicator function provides an improved target object localization, which is very useful for alleviating the problem of background clutter. In order to verify such effectiveness of the target model using the indicator function, we performed an extensive comparative study using five carefully selected video sequences and three different kernel sizes. For all the compared representative previous algorithms, which deal with the problem of background clutter, it has been observed that the proposed target model significantly improves the performance of the tracking robustness as well as the accuracy with a negligible computational complexity increment. The improved tracking accuracy gain reached up to 56.6% and the maximum improvement of tracking robustness was 40% at the cost of less than 0.47% of tracking time increment.

Finally, although the proposed indicator function was employed only for the construction of a target representation model in this paper, it is also expected to be very useful in dealing with various problems of mean-shift tracking such as the scale estimation of target objects, target model updating, and occlusion detection, *etc*.

## Figures and Tables

**Table 1.** Features of the selected video sequences.

**Video**	**Size (Numbers of Frames)**	**Kernel**	**Scale Change**	**Deform**	**Occlusion**
Baseball-1	960 × 540 (181)	139 × 149	mid	large	no
BaseBall-2	729 × 480 (215)	33 × 61	large	large	no
Campus	768 × 576 (209)	43 × 93	small	mid	mid
EgTest05	640 × 480 (199)	93 × 47	no	no	large
Bike	320 × 240 (48)	23 × 33	no	no	no

**Table 2.** Tracking results before and after applying the proposed indicator function by Equation (11). (F) means tracking failure and the number in brackets represents the improvement ratio of tracking accuracy caused by applying the proposed indicator function.

**Seq. Name**	**Kernel Size**	**Previous Algorithms**				**Proposed Algorithms (Using Indicator Function)**			
		**A1 [2]**	**A2 [12]**	**A3 [14]**	**A4 [13]**	**P1**	**P2**	**P3**	**P4**
Baseball-1	small	21.1	**5.7**	24.6	6.1	19.1 [9.3]	**5.7** [0.0]	23.8 [3.0]	5.8 [6.1]
	normal	33.7	4.7	30.4	4.1	21.1 [37.3]	**3.5** [24.9]	26.9 [11.5]	4.0 [1.0]
	large	45.9	9.9	43.4	6.2	26.7 [41.9]	8.9 [10.2]	37.1 [14.5]	**6.0** [2.5]
	Avg.	33.6	6.8	32.8	5.5	22.3 [33.6]	6.0 [11.8]	29.3 [10.7]	**5.3** [3.6]
Baseball-2	small	25.4 (F)	**3.6**	40.5 (F)	4.4	4.5 [82.4]	3.9 [−8.5]	4.9 [87.9]	4.4 [0.0]
	normal	86.2 (F)	3.7	91.9 (F)	**3.3**	5.8 [93.3]	3.5 [5.9]	7.5 [91.8]	3.4 [−3.7]
	large	90.6 (F)	4.7	95.3 (F)	4.7	7.5 [91.8]	**3.9** [15.3]	11.6 [87.8]	4.4 [5.4]
	Avg.	67.4	4	75.9	4.1	5.9 [91.2]	**3.8** [5.0]	8.0 [89.5]	4.1 [0.0]
Campus	small	64.5 (F)	8.2	66.1 (F)	8.6	8.7 [86.6]	7.1 [12.5]	9.3 [85.9]	**6.7** [22.0]
	normal	79.9 (F)	5.8	78.8 (F)	6.8	13.6 [82.9]	**4.2** [26.6]	55.5 (F) [29.6]	6.4 [6.2]
	large	90.0 (F)	17.1	87.9 (F)	11.5	18.2 [79.8]	**7.4** [56.6]	58.5 (F) [33.5]	10.5 [8.0]
	Avg.	78.1	10.3	77.6	9	13.5 [82.7]	**6.3** [38.8]	41.1 [47.0]	7.9 [12.2]
EgTest05	small	11.8	7.5	13.9	7.5	10.6 [10.4]	6.0 [19.7]	11.1 [20.6]	**4.4** [41.2]
	normal	21.7	8.5	23.9	7.3	14.0 [35.3]	6.4 [25.4]	15.6 [34.9]	**5.1** [30.3]
	large	45.0	11.9	141.0 (F)	9.6	25.3 [43.8]	9.4 [21.5]	32.6 [76.9]	**8.0** [16.7]
	Avg.	26.2	9.3	59.6	8.1	16.6 [36.6]	7.3 [21.5]	19.8 [66.8]	**5.8** [28.4]
Bike	small	89.7 (F)	45.8 (F)	94.4 (F)	3.7	81.2 (F) [9.5]	**3.0** [93.5]	89.1 (F) [5.6]	3.1 [16.3]
	normal	88.1 (F)	77.0 (F)	85.3 (F)	4.9	74.4 (F) [15.5]	**2.4** [96.9]	74.3 (F) [13.0]	2.9 [39.7]
	large	88.4 (F)	76.9 (F)	84.9 (F)	104.1 (F)	77.3 (F) [12.5]	**2.3** [97.0]	4.7 [94.5]	2.9 [97.2]
	Avg.	88.7	66.6	88.2	37.6	77.6 [12.5]	**2.6** [96.1]	56.0 [36.5]	3.0 [92.0]
Tracking Failure Rate		60.0	20.0	66.7	6.7	20.0	0.0	26.7	0.0

**Table 3.** RI and AI ratio of each proposed scheme.

	**P1**	**P2**	**P3**	**P4**
**Robustness Improvement**	40	20	40	6.7
**Average of Accuracy Improvement**	29.7	17.5	16.9	13.7

**Table 4.** Average execution time for each processing stage of tracking algorithms. (unit: millisecond).

**Processing Step**	**Previous Algorithms**				**Proposed Algorithms (Using Indicator Function)**			
	**A1 [2]**	**A2 [12]**	**A3 [14]**	**A4 [13]**	**P1**	**P2**	**P3**	**P4**
Avg. time for target model construction	0.74	0.75	1.05	0.74	1.13	1.35	1.51	1.37
Avg.time for target candidate construction per iteration (TCC)	0.31	0.33	1.04	0.31	0.32	0.33	1.04	0.33
Avg.time for new centroid computation per iteration (NCC)	0.48	0.49	0.47	0.48	0.48	0.48	0.48	0.48
Avg. number of iteration per frame (I)	2.15	2.06	2.18	2.04	2.12	2.04	2.14	2.04
Avg. execution time for tracking per frame: (TCC + NCC) × I	1.72	1.70	3.31	1.64	1.72	1.67	3.27	1.66
